# Glucagon-like peptide-1 receptor analog use is associated with reduced thromboembolic events compared with dipeptidyl peptidase-4 inhibitors in rheumatoid arthritis patients: A global retrospective cohort study

**DOI:** 10.1007/s10067-025-07709-0

**Published:** 2025-09-27

**Authors:** Qi Wang, Donald D. Anthony

**Affiliations:** 1 Devision of Rheumatology, MetroHealth Medical Center, Cleveland, OH USA; 2 Department of Medicine, Case Western Reserve University School of Medicine, Cleveland, OH USA; 3 Cleveland Veterans Affairs Medical Center, Cleveland, OH USA

**Keywords:** Diabetes mellitus, GLP-1 analog, Rheumatoid arthritis, Thrombotic events

## Abstract

**Objective:**

Rheumatoid arthritis (RA) is an inflammatory disease associated with thromboembolic events. Glucagon-like peptide-1 (GLP-1) analogs, which are used in type 2 diabetes mellitus (T2DM), have shown potential anti-inflammatory effects, but their role in thrombotic events in RA is less clear. This study compares GLP-1 analogs to dipeptidyl peptidase 4 inhibitors (DPP4i) regarding thromboembolic events in RA.

**Methods:**

We performed a retrospective cohort study using TriNetX database to evaluate adult patients who carried the diagnosis of RA from January 1, 2006 to December 1, 2024 with co-existing T2DM and who initiated GLP-1 analogs or DPP4i after diagnosis of RA and T2DM. Patients were divided into two cohorts (GLP-1 vs. DPP4i), and the primary outcome was all thromboembolic events (cerebral infarction, myocardial infarction [MI], deep vein thrombosis [DVT], pulmonary embolism [PE]) within 5 years after GLP-1 analog or DPP4i start. Secondary outcomes included individual events, arterial and venous thrombosis, and all-cause mortality. Propensity score matching adjusted for demographic, clinical, and treatment variables.

**Results:**

We analyzed 41,153 patients, including 25,425 GLP-1 analog and 15,728 DPP4i users, and compared 8,697 matched patients in each group. The median age at medication start was 65 years old, with 70% females. GLP-1 analog users had a 24% lower risk of all thrombotic events (hazard ratio [HR], 0.76 [95% CI: 0.70, 0.83]; *p* < 0.0001), as well as reduced risks of individual events and an overall lower mortality.

**Conclusion:**

Compared to DPP4i, GLP-1 analogs may lower the risk of thromboembolic events and reduce all-cause mortality in RA patients with T2DM.

**Table:** 

**Key Points** • *The study identified significantly reduced risks in both arterial and venous thrombosis in RA patients receiving GLP-1 analogs compared with DPP4i*. • *RA patients who received GLP-1 analogs had lower all-cause mortality compared to those receiving DPP4i*. • *These findings support potential dual benefit of GLP-1 analogs in reducing inflammation and thrombosis in RA patients with diabetes*.

**Supplementary Information:**

The online version contains supplementary material available at 10.1007/s10067-025-07709-0.

## Introduction

Glucagon-like peptide-1 (GLP-1) analogs, widely used in type 2 diabetes mellitus (T2DM), have shown reduced risks of cardiovascular events, stroke, thrombotic events and all-cause mortality in populations with diabetes and obesity in previous studies [1, 2]. In contrast, DPP4i have not shown cardioprotective benefits, while they are effective in control of T2DM [1]. It is thought that this is likely via anti-inflammatory activity [1], and by attenuating platelet aggregation [2].

Rheumatoid arthritis (RA) is associated with higher risk of cardiovascular events [3] and thromboembolic events [4]. Whether the same benefit is achievable in persons with RA has been less clear. A recent population-based cohort study showed GLP-1 analog use reduced risk of stroke, major adverse cardiovascular events (MACE), and all-cause mortality [5]. Notably, in the RA subgroup, which comprised 38% of the total study population, GLP-1 analog use was also associated with a significant reduction in MACE and all-cause mortality. However, the impact on thromboembolic events, particularly deep vein thrombosis (DVT) and pulmonary embolism (PE), remains unclear. Here we evaluated a real-world cohort of RA and T2DM, focusing on arterial and venous thrombosis outcomes.

## Material and methods

### Study design and participants

In this study, we used data from TriNetX network. TriNetX is a network that contains de-identified data including demographics, diagnosis, medications, and laboratory results from more than 70 participating healthcare organizations (HCOs) across the world. This database is under strict Health Insurance Portability and Accountability Act (HIPAA) policy. This study was exempt from an institutional review board (IRB) or ethics committee, as it did not involve human subjects or identifiable private information.

We enrolled adult patients (aged ≥ 18) carrying the diagnosis of RA between January 1, 2006, and December 1, 2024, with co-existing T2DM who received GLP-1 analogs or DPP4i after the diagnosis of RA and T2DM. Diagnoses were identified using International Classification of Diseases, Tenth Revision (ICD-10) codes in TriNetX (Supplementary Table 1). The index event was defined as the initiation of GLP-1 analogs or DPP4i following the diagnosis of RA.

The GLP-1 analog cohort included patients who received GLP-1 analogs after the diagnosis of RA and T2DM, and patients who received DPP4i were excluded from this group. Similarly, the DPP4i cohort included patients who received DPP4i after the diagnosis of RA and T2DM, and patients who received GLP-1 analogs were excluded from this group. In both cohorts, patients who had previous cerebral infarction, MI, DVT, or PE prior to the diagnosis of RA were also excluded.

Both cohorts were followed over 5 years for all thrombotic events including stroke, MI, DVT and PE, individual thrombotic events, arterial thrombotic events including stroke and MI, and venous thrombotic events including DVT and PE.

### Outcome definition

The primary outcome was defined as patients developing thromboembolic events including arterial thrombosis, cerebral infarction, MI, and venous thrombosis including DVT and PE. Secondary outcomes included patients developing cerebral infarction, MI, DVT, PE, arterial thrombosis which was defined as the combination of cerebral infarction and MI, venous thrombosis which was defined as the combination of DVT and PE, and all-cause mortality.

### Statistical analysis

Propensity score matching (PSM) was used to balance potential confounding factors in a 1:1 ratio. Variables including age, age at index event (GLP-1 analogs vs. DPP4i start), gender, race, body max index (BMI), hemoglobin A1c (HbA1c), tobacco use, use of other diabetic medications, cardiovascular medications, anticoagulation and Disease-Modifying Antirheumatic Drugs (DMARDs) (Supplementary Table 2). Standardized mean differences (SMD) below 0.1 indicates that the variable is distributed in balance between the two cohorts. All the data were obtained and analyzed using the TriNetX platform. We used Kaplan–Meier analysis and log-rank test to compare outcomes in two groups and cox proportional hazards analysis to evaluate the association between GLP-1 analogs use and the outcomes of interest. Two-tailed p-values below 0.05 were considered statistically significant.

## Results

We analyzed 41,153 patients diagnosed with RA and T2DM, including 25,425 patients treated with GLP-1 analogs and 15,728 patients treated with DPP4i. After propensity score matching, 8,697 patients per group were compared. Baseline characteristics were well-balanced with SMD values below 0.10 except for BMI (35.1 ± 8.0 vs. 32.0 ± 7.8, SMD = 0.39). The mean ages at medication start were 65 years with 70% being female, and the mean HbA1c was 7.6. The prevalence of pre-existing co-morbidities was similar between the cohorts, as was the use of aspirin, clopidogrel, other diabetic medications, cardiovascular medications, and DMARDs (Table 1).

**Table 1:** Patient baseline characteristics after propensity score matching

Characteristic Name	GLP-1 analog	DPP4i	*P*-value	Standardized mean difference (SMD)
	(*n*=8,697)	(*n*=8,697)		
**Basic demographics**				
Age, mean, years	69.7 +/- 9.8	69.9 +/- 11.1	0.354	0.014
Age at index, mean, years	64.8+/−10.2	65.0+/−11.8	0.314	0.015
White	5,203 (59.8*)	5,119 (58.9)	0.195	0.020
Black or African American	1,553 (17.9)	1,631 (18.8)	0.126	0.023
Hispanic or Latino	970 (11.2)	967 (11.1)	0.942	0.001
Asian	404 (4.6)	398 (4.6)	0.828	0.003
Female	6,099 (70.1)	6,081 (69.9)	0.766	0.005
Tobacco use	431 (5.0)	441 (5.1)	0.728	0.005
BMI, mean, kg/m2	35.1 +/- 8.0	32.0 +/- 7.8	<0.001	0.389
**Underlying comorbidities**				
Hypertensive diseases	6,591 (75.8)	6,620 (76.1)	0.607	0.008
Atrial fibrillation and flutter	975 (11.2)	975 (11.2)	1	<0.001
Heart failure	1,400 (16.1)	1,429 (16.4)	0.551	0.009
Disorders of lipoprotein metabolism and other lipidemias	5,895 (67.8)	5,897 (67.8)	0.974	<0.001
Overweight, obesity and other hyperalimentation	3,558 (40.9)	3,617 (41.6)	0.363	0.014
Chronic kidney disease (CKD)	1,999 (23.0)	2,037 (23.4)	0.495	0.010
**Pertinent lab data**				
Hemoglobin A1c, mean	7.6 +/- 1.9	7.6 +/- 1.8	0.720	0.007
**Use of diabetes medications**				
Metformin	4,747 (54.6)	4,717 (54.2)	0.648	0.007
Insulin	4,028 (46.3)	4,104 (47.2)	0.248	0.018
Glipizide	1,330 (15.3)	1,308 (15.0)	0.642	0.007
Glimepiride	877 (10.1)	870 (10.0)	0.860	0.003
Glyburide	373 (4.3)	376 (4.3)	0.911	0.002
Empagliflozin	678 (7.8)	674 (7.7)	0.910	0.002
Dapagliflozin	357 (4.1)	350 (4.0)	0.788	0.004
Canagliflozin	190 (2.2)	192 (2.2)	0.918	0.002
Repaglinide	109 (1.3)	102 (1.2)	0.628	0.007
Pioglitazone	555 (6.4)	556 (6.4)	0.975	<0.001
Rosiglitazone	58 (0.7)	57 (0.7)	0.925	0.001
**Use of cardiovascular medications**				
Aspirin	3,370 (38.7)	3,418 (39.3)	0.456	0.011
Clopidogrel	776 (8.9)	770 (8.9)	0.873	0.002
Atorvastatin	3,372 (38.8)	3,438 (39.5)	0.305	0.016
Rosuvastatin	1,214 (14.0)	1,251 (14.4)	0.421	0.012
Pravastatin	902 (10.4)	921 (10.6)	0.638	0.007
Simvastatin	1,328 (15.3)	1,338 (15.4)	0.833	0.003
Angiotensin-converting enzyme inhibitors (ACEI)	3,224 (37.1)	3,206 (36.9)	0.777	0.004
Angiotensin II receptor blockers (ARB)	2,747 (31.6)	2,769 (31.8)	0.720	0.005
Calcium Channel Blockers (CCB)	3,175 (36.5)	3,260 (37.5)	0.182	0.020
**Use of immunomodulator/immunosuppressant**				
Hydroxychloroquine	1,490 (17.1)	1,506 (17.3)	0.748	0.005
Azathioprine	213 (2.4)	212 (2.4)	0.961	0.001
Mycophenolate mofetil	207 (2.4)	211 (2.4)	0.843	0.003
Mycophenolic acid	75 (0.9)	76 (0.9)	0.935	0.001
Methotrexate	1,711 (19.7)	1,708 (19.6)	0.954	0.001
Infliximab	173 (2.0)	157 (1.8)	0.374	0.013
Leflunomide	568 (6.5)	544 (6.3)	0.457	0.011
Adalimumab	502 (5.8)	480 (5.5)	0.470	0.011
Etanercept	394 (4.5)	385 (4.4)	0.741	0.005
Abatacept	191 (2.2)	190 (2.2)	0.959	0.001
Tocilizumab	112 (1.3)	107 (1.2)	0.734	0.005
Tofacitinib	193 (2.2)	172 (2.0)	0.267	0.017
Upadacitinib	43 (0.5)	34 (0.4)	0.304	0.016
Certolizumab	0	0		N/A
Golimumab	62 (0.7)	69 (0.8)	0.539	0.009
Sulfasalazine	585 (6.7)	538 (6.2)	0.147	0.022
Rituximab	136 (1.6)	134 (1.5)	0.902	0.002
Prednisone	3.476 (40.0)	3,489 (40.1)	0.841	0.003
Methylprednisolone	2,869 (33.0)	2,891 (33.2)	0.723	0.005
**Use of anticoagulation**				
Heparin	2,078 (23.9)	2,130 (24.5)	0.357	0.014
Enoxaparin	1,686 (19.4)	1,730 (19.9)	0.401	0.013
Warfarin	438 (5.0)	419 (4.8)	0.506	0.010
Apixaban	533 (6.1)	528 (6.1)	0.874	0.002
Rivaroxaban	297 (3.4)	309 (3.6)	0.620	0.008

*Values in parentheses are percentages

### Primary outcome

**Fig. 1:**
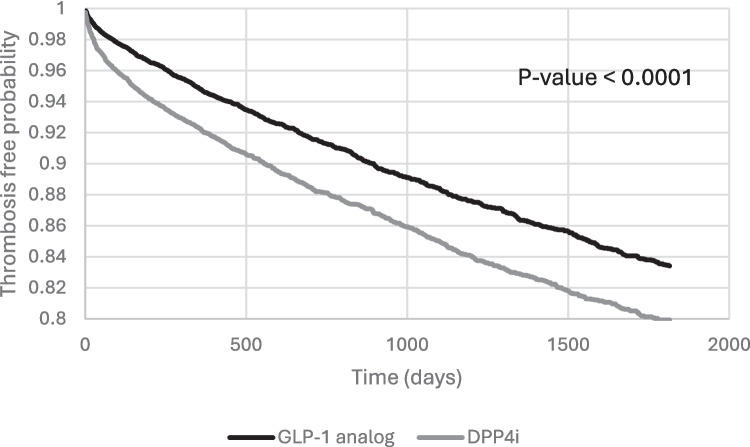
Kaplan–Meier survival curve of thrombosis-free probability over 5 years

Over the follow up of 5 years, 958 patients in the GLP-1 analog cohort developed thrombotic events including stroke, MI, DVT or PE, while 1,165 patients in DPP4i cohort developed thrombotic events. Kaplan–Meier survival analysis demonstrated a higher thrombosis free probability over time in GLP-1 analog group (Fig. 1). Cox proportional hazards analysis revealed that GLP-1 analogs were associated with a 24% lower risk of all thrombotic events (Hazard ratio (HR), 0.76 [95% CI: 0.70–0.83]; *p* < 0.0001) compared to DPP4i (Table 2).

**Table 2:** Cox proportional hazards analysis for the association between the use of GLP-1 analogs vs DPP4i and patient outcomes

Outcomes	GLP-1 analogs		DPP4i		Hazard ratio (95% CI)	*P*-value (Log-rank)
	At risk patients	Cases	At risk patients	Cases		
*Primary outcome*						
All thromboembolic events	8,697	958	8,697	1,165	0.76 (0.70, 0.83)	< 0.0001
*Secondary outcomes*						
Cerebral infarction	8,697	389	8,697	478	0.76 (0.67, 0.87)	< 0.0001
Myocardial infarction	8,697	399	8,697	516	0.72 (0.63, 0.82)	< 0.0001
Deep vein thrombosis	8,697	147	8,697	196	0.70 (0.57, 0.87)	0.001
Pulmonary embolism	8,697	192	8,697	201	0.90 (0.74, 1.09)	0.282
All arterial thrombosis	8,697	726	8,697	911	0.74 (0.67, 0.81)	< 0.0001
All venous thrombosis	8,697	302	8,697	354	0.80 (0.68, 0.93)	0.004
All-cause mortality	8,697	606	8,697	1,011	0.56 (0.50, 0.62)	< 0.0001

Shown are hazard ratio (5%, 95% confidence interval) and log rank *p* value for each comparison between groups

### Secondary outcomes

Over the 5-year follow up, the GLP-1 analog cohorts developed less events compared with DPP4i cohort: cerebral infarction (389 vs. 478, HR 0.76 [95% CI: 0.67, 0.87]; *p* < 0.0001), MI (399 vs. 516, HR 0.72 [95% CI: 0.63, 0.82]; *p* < 0.0001), DVT (147 vs. 169, HR 0.70 [95% CI: 0.57, 0.87]; *p* = 0.001), PE (192 vs. 201 HR 0.90 [95% CI: 0.74, 1.09]; *p* = 0.282). GLP-1 analogs were associated with lower risk of all arterial thrombosis (HR, 0.74, [95% CI: 0.67, 0.81]; *p* < 0.0001) and venous thrombosis (HR, 0.80 [95% CI: 0.68, 0.93]; *p* = 0.004), as well as an overall decreased all-cause mortality (HR 0.56 [95% CI: 0.50, 0.62]; *p* < 0.0001) (Table 2). Although the reduction in PE was not statistically significant, DVT and overall venous thrombosis were significantly reduced, with PE showing a trend toward lower risk.

## Discussion

RA is one of the most prevalent chronic inflammatory autoimmune diseases with joints being predominately involved. It carries increased risk for cardiovascular disease [3], as well as venous thrombotic events [4]. Though the exact etiology of RA associated thrombosis is not completely clear, systemic inflammation leading to endothelial dysfunction and hypercoagulation are thought to play a role [6]. In RA, early endothelial dysfunction is driven by inflammatory cytokines including tumor necrosis factor-α (TNF-α), interleukin (IL)−1, and is reflected by increased C-reactive protein (CRP). This leads to increased expression of adhesion molecules and enhanced leukocyte and platelet adhesion to the endothelium, as well as increased permeability, and plasminogen activator inhibitor-1 (PAI-1), predisposing to thrombosis and disease progression [7]. Furthermore, RA can upregulate a hypercoagulability state by increasing inflammatory markers like fibrinogen, D-dimer, cytokines (IL-6, IL-8, TNF), and CRP, which drive coagulation at both extravascular and intravascular sites [6]. Activated platelets and microparticles (MP), influenced by oxidative stress and autoimmunity, can also enhance thrombin generation and platelet activation [8].

The cardiovascular protective and anti-thrombotic effects of GLP-1 analogs can be attributed to multiple mechanisms. Research has shown that GLP-1 analogs reduce inflammation through various pathways, including decreasing the production of reactive oxygen species [9], and lowering levels of inflammatory cytokines such as TNF-α, IL-1β, and IL-6 in mononuclear cells [10]. Additionally, they inhibit nuclear factor (NF)-κB binding in mononuclear blood cells, which may contribute to their anti-atherogenic effects [11]. GLP-1 analogs also show efficacy in reducing Thromboxane A2 (TXA2), a pro-inflammatory mediator involved in platelet activation [2]. Moreover, as obesity is linked to chronic inflammation originating from adipose tissue [12] and GLP-1 analogs are effective in promoting weight loss [13] thus reduce inflammation. DPP4i, which also act on incretin-based pathways like GLP-1 analogs, have not been shown to provide significant cardiovascular protection or anti-thrombotic effects [1]. While they share a similar pathway and demonstrate some anti-inflammatory properties [14], they lack evidence of effects on inhibiting endothelial function [15] and weight loss. Further research is needed to determine whether the anti-inflammatory effects of GLP-1 analogs are more pronounced compared to DPP4i, though the identified effects on TNF, IL1B and IL6 do provide overlap with those pathways engaged in RA, and provide an area of focus for future studies.

While a recent meta-analysis by Liu et al. [16] reported that long-term use of GLP-1 analogs was associated with increased risk of DVT in general population with T2DM or other metabolic syndromes, contrasting with our findings, our study focused on a distinct population with RA who carry chronic systemic inflammation, and have unique pathophysiological mechanisms including endothelial dysfunction and upregulated inflammatory cytokines which are known to increase thrombotic risk. In this context, the anti-inflammatory effects of GLP-1 analogs may confer protective benefits in RA populations and reduce thrombotic risk.

This study has several limitations. First, as a retrospective analysis using the TriNetX database, patient identification relied on ICD-10 codes rather than classification criteria, introducing risks of misdiagnosis and overdiagnosis. Second, despite propensity score matching, unmeasured confounders, such as duration and dosage of glucocorticoid use, the length of tobacco use, family history, and medication compliance may remain. Third, even after matching, the BMI in the GLP-1 analog group was significantly higher than in the DPP4i group, likely because patients with higher BMI are more inclined to use GLP-1 analogs. Nonetheless, the GLP-1 analog cohort exhibited a lower risk of thrombotic events despite starting with a higher BMI. Fourth, some factors could not be quantified using the TriNetX database, such as atherosclerotic cardiovascular disease (ASCVD) score. Fifth, due to the limitations of the database, we were unable to include RA-specific information such as disease duration, disease activity including the Disease Activity Score in 28 joints (DAS28) and the Clinical Disease Activity Index (CDAI), and seropositivity defined by RF and anti-cyclic citrullinated peptide antibodies (anti-CCP). Whether serologic status influences the reduced thrombotic risk associated with GLP-1 analog use warrants further investigation. Sixth, DMARD exposure was identified but RA duration was unavailable. Seventh, although TriNetX collects laboratory data such as CRP and erythrocyte sedimentation rate (ESR), we were unable to access individual patient charts to verify the etiologies of abnormal results and variability precluded their use in matching. Eighth, we did not compare the changes in HbA1c between the two groups because these levels were measured multiple and differing times throughout the study period, preventing standardized comparisons between groups.

## Conclusion

This study demonstrated that GLP-1 analog use is associated with decreased risk of all types of thrombotic events including arterial as well as venous thrombosis, with decreased all-cause mortality compared with DPP4i. These findings offer valuable insights into the potential role of GLP-1 analogs in managing RA patients with co-existing diabetes and provide perspective on inflammatory pathways that may be particularly relevant in persons with RA and T2DM.

## Supplementary Information

Below is the link to the electronic supplementary material.

Supplementary file1 (DOCX 22 KB)
