# Supplementary material for: Glucagon-like peptide-1 receptor analog use is associated with reduced thromboembolic events compared with dipeptidyl peptidase-4 inhibitors in rheumatoid arthritis patients: A global retrospective cohort study
Source: Clin Rheumatol. 2025 Sep 27;44(11):4479–85. doi: 10.1007/s10067-025-07709-0 (PMC12568834; doi:10.1007/s10067-025-07709-0)
Supplement: Supplementary file 1 — Supplementary file1 (DOCX 22 KB) [file 10067_2025_7709_MOESM1_ESM.docx]

**Supplementary Table 1. Disease, medication, and outcome definitions**

| \| Disease or medication \| ICD-10 codes or TriNetX codes (RxNorm, ATC, CPT, SNOMED) \| \| --- \| --- \| \| Type 2 diabetes mellitus \| E11 \| \| Glucagon-like peptide-1 (GLP-1) analogues \| A10BJ \| \| Dipeptidyl peptidase 4 (DPP-4) inhibitors \| A10BH \| \| Rheumatoid arthritis \| Rheumatoid arthritis, unspecified (M06.9), Other rheumatoid arthritis (M06), Rheumatoid arthritis with rheumatoid factor (M05) \| \| All-cause mortality \| Deceased \| \| Myocardial infarction \| Acute myocardial infarction (I21), Subsequent ST elevation (STEMI) and non-ST elevation (NSTEMI) myocardial infarction (I22) \| \| Cerebral infarction \| I63 \| \| Pulmonary embolism \| I26 \| \| Deep vein thrombosis \| Acute embolism and thrombosis of unspecified deep veins of lower extremity (I82.40) \| |
| --- | --- | --- | --- | --- | --- | --- | --- | --- | --- | --- | --- | --- | --- | --- | --- | --- | --- | --- | --- | --- |

**Supplementary Table 2. Variables used in the propensity score matching model**

| **Characteristic name** | **Codes** |
| --- | --- |
| **Basic demographic** |  |
| Age, mean, years | Built in variable |
| White | Built in variable |
| Black or African American | Built in variable |
| Hispanic or Latino | Built in variable |
| Asian | Built in variable |
| Female | Built in variable |
|  |  |
| **Underlying comorbidities** |  |
| Hypertensive diseases | I10-I1A |
| Atrial fibrillation and flutter | I48 |
| Heart failure | I50 |
| Disorders of lipoprotein metabolism and other lipidemias | E78 |
| Overweight, obesity and other hyperalimentation | E65-E68 |
| Chronic kidney disease (CKD) | N18 |
| **Pertinent lab data** |  |
| Hemoglobin A1c/Hemoglobin.total in Blood | 9037 |
| **Use of diabetes medications** |  |
| Metformin | 6809 |
| Insulin | HS501 |
| Glipizide | 4821 |
| Glimepiride | 25789 |
| Glyburide | 4815 |
| Empagliflozin | 1545653 |
| Dapagliflozin | 1488564 |
| Canagliflozin | 1373458 |
| Repaglinide | 73044 |
| Pioglitazone | 33738 |
| Rosiglitazone | 84108 |
| **Use of cardiovascular medications** |  |
| Aspirin | 1191 |
| Clopidogrel | 32968 |
| Atorvastatin | 83367 |
| Rosuvastatin | 301542 |
| Pravastatin | 42463 |
| Simvastatin | 36567 |
| Angiotensin-converting enzyme inhibitors (ACEI) | CV800 |
| Angiotensin II receptor blockers (ARB) | CV805 |
| Calcium Channel Blockers (CCB) | CV200 |
| **Use of immunomodulator/immunosuppressant** |  |
| Hydroxychloroquine | 5521 |
| Azathioprine | 1256 |
| Mycophenolate mofetil | 68149 |
| Mycophenolic acid | 7145 |
| Methotrexate | 6851 |
| Infliximab | 191831 |
| Leflunomide | 27169 |
| Adalimumab | 327361 |
| Etanercept | 214555 |
| Abatacept | 614391 |
| Tocilizumab | 612865 |
| Tofacitinib | 1357536 |
| Upadacitinib | 2196092 |
| Certolizumab | OMOP5175190 |
| Golimumab | 819300 |
| Sulfasalazine | 9524 |
| Rituximab | 121191 |
| Prednisone | 8640 |
| Methylprednisolone | 6902 |
| **Use of anticoagulation** |  |
| Heparin | 5224 |
| Enoxaparin | 67108 |
| Warfarin | 11289 |
| Apixaban | 1364430 |
| Rivaroxaban | 1114195 |

**Supplementary Table 3. List of abbreviations**

| **Abbreviation** | **Full term** |
| --- | --- |
| ACEI | Angiotensin-converting enzyme inhibitors |
| Anti-CCP | Anti-cyclic citrullinated peptide antibodies |
| ARB | Angiotensin II receptor blockers |
| ASCVD | Atherosclerotic cardiovascular disease |
| BMI | Body mass index |
| CCB | Calcium channel blockers |
| CDAI | Clinical Disease Activity Index |
| CI | Confidence interval |
| CRP | C-reactive protein |
| DAS28 | Disease Activity Score in 28 joints |
| DMARDs | Disease-modifying antirheumatic drugs |
| DPP4i | Dipeptidyl peptidase-4 inhibitors |
| DVT | Deep vein thrombosis |
| ESR | Erythrocyte sedimentation rate |
| GLP-1 | Glucagon-like peptide-1 |
| HbA1c | Hemoglobin A1c |
| HCOs | Healthcare organizations |
| HIPAA | Health Insurance Portability and Accountability Act |
| HR | Hazard ratio |
| ICD-10 | International Classification of Diseases, 10th Revision |
| IL | Interleukin |
| IRB | Institutional Review Board |
| MACE | Major adverse cardiovascular events |
| MI | Myocardial infarction |
| MP | Microparticles |
| NF-κB | Nuclear factor kappa B |
| PAI-1 | Plasminogen activator inhibitor-1 |
| PE | Pulmonary embolism |
| PSM | Propensity score matching |
| RA | Rheumatoid arthritis |
| SMD | Standardized mean difference |
| T2DM | Type 2 diabetes mellitus |
| TNF-α | Tumor necrosis factor-alpha |
| TXA2 | Thromboxane A2 |
